# Occurrence of Stress and Burnout Among Nurses Employed in a Psychiatric Hospital and a Somatic Hospital—A Comparative Analysis (Nursing Workload KEGA č. 011KU-4/2024)

**DOI:** 10.3390/healthcare12232443

**Published:** 2024-12-04

**Authors:** Katarzyna Tomaszewska, Krystyna Kowalczuk, Helena Kadučáková, Mária Lehotská, Katalina Papp, Bożena Majchrowicz

**Affiliations:** 1 Department of Nursing, Institute of Health Protection, The Bronisław Markiewicz Academy of Applied Sciences, 37-500 Jarosław, Poland; 2 Department of Integrated Medical Care, Medical University of Białystok, 15-222 Białystok, Poland; krystyna.kowalczuk@umb.edu.pl; 3 Department of Nursing, Faculty of Health Sciences, Catholic University in Ružomberok, 034 01 Ružomberok, Slovakia; helena.kaducakova@ku.sk (H.K.); maria.lehotska@ku.sk (M.L.); 4 Department of Health Care Sciences, Faculty of Humanities, Tomas Bata University in Zlin, 760 01 Zlín, Czech Republic; 5 Faculty of Health Sciences, University of Debrecen, 4032 Debrecen, Hungary; papp.katalin@etk.unideb.hu; 6 Department of Nursing, Institute of Health Protection, State Academy of Applied Sciences, 37-700 Przemyśl, Poland; bozena.majchrowicz01@gmail.com

**Keywords:** nurse, stress, occupational burnout

## Abstract

Work-related stress has been linked to various negative outcomes among healthcare professionals. For nurses, stress can arise from numerous sources, including their interactions with patients. It is often perceived that nurses working in psychiatric hospitals experience greater stress and occupational burnout compared to nurses working in somatic hospitals. However, there is limited research addressing this specific issue. To bridge this gap, a study was conducted to compare the stress levels of nurses working in a psychiatric hospital and a somatic hospital within the same city. **Background/Objectives:** The aim of this paper was to report on the prevalence of stress and burnout among surveyed nurses employed in a somatic hospital and in a psychiatric hospital. **Methods:** The study group consisted of a total of 379 nurses—189 employed at a somatic hospital and 190 employed at a psychiatric hospital. The primary test used for statistical analyses was the nonparametric Mann–Whitney U test for assessing differences. Additionally, correlations between ordinal or quantitative variables were analyzed using Spearman’s rho coefficient. **Results:** Among respondents working at a somatic hospital, the average levels of occupational burnout, emotional exhaustion, depersonalization, and sense of personal accomplishment were moderate. Similar results were observed among respondents employed at a psychiatric hospital. **Conclusions:** The workplace does not significantly differentiate professional burnout or coping strategies among the nurses surveyed. Among nurses working in hospitals for somatic patients, levels of depersonalization, turning to religion, and seeking support increase with age and seniority. In contrast, psychiatric nurses show higher levels of emotional exhaustion and overall MBI burnout as they age.

## 1. Introduction

Nurses represent the largest group of healthcare professionals, primarily tasked with providing direct patient care. Regardless of the clinical setting, the demands of healthcare delivery can be highly stressful and pose risks to their personal safety. These challenges are influenced not only by the workplace, such as somatic hospitals or psychiatric facilities, but also by the patients’ conditions [1]. Healthcare staff face numerous challenges while ensuring patient safety and delivering high-quality care. The demands imposed by employers and patients often subject nurses to intense stress and constant pressure [2].

A general hospital, also referred to as a somatic hospital, provides care for patients with somatic diseases [3]. These patients often present with physical symptoms such as pain, organ dysfunction, or general weakness [4,5], sometimes rooted in psychological disorders, prolonged stress, or fear for their lives [6]. In such hospitals, nurses may also encounter aggressive behavior, especially among patients experiencing drug or alcohol withdrawal or those with mental health issues, often directed toward nurses physically and verbally [7].

Conversely, psychiatric nurses often face heightened stress levels due to the nature of their work, which involves caring for individuals with suicidal tendencies, aggression, or threats of violence. This leads to greater emotional strain compared to nurses in other specialties [1]. Psychiatric nurses also use themselves as therapeutic tools, facing increased professional demands and stress, particularly when caring for patients with repeated or prolonged hospitalizations. Additionally, they often manage acutely psychotic, aggressive, or suicidal individuals [8,9].

Psychiatric units present unique challenges, exposing nurses to psychological stress, occupational burnout, depression, anxiety, and distress [10]. These units require managing acute mental health crises and emotionally demanding patient interactions [11,12]. Chronic exposure to such stressors can lead to burnout, characterized by emotional exhaustion, cynicism, and a diminished sense of personal achievement [13,14].

Stress is a topic of interest to many researchers, defined variably as both a mobilizing force for daily activities and a negative factor impacting physical and mental health. Stress arises when individuals perceive situations as difficult to manage or threatening. Its effects depend on the nature of the stressors, which may stem from work or living environments, and can manifest in symptoms like anxiety, reduced attention, sweating, palpitations, or hand tremors. Individuals may resort to alcohol or psychotropic substances to cope [15]. Prolonged occupational stress can impair daily functioning, reduce care quality, and lead to burnout [16]. Hospitals, as workplaces, have long been recognized as highly stressful environments, particularly for nurses [17].

According to Rafiei et al., occupational stress results from various factors, including personnel, work environment, and cultural conditions. It manifests in responses to job demands exceeding one’s capacity [18,19]. Job satisfaction among nurses encompasses remuneration, task accomplishment, handling difficult situations, and teamwork dynamics [20]. Imbalances in these areas can contribute to burnout syndrome, described by Suleiman-Martos et al. as physical, emotional, and mental exhaustion caused by chronic stress [21]. The intense emotional demands of nursing predispose many to depression and anxiety [22,23].

Occupational burnout is common in healthcare, especially among nurses, who engage in close interpersonal relationships with patients [24]. Defined as a reaction to chronic workplace stress, burnout has been included in the World Health Organization’s International Classification of Diseases [25]. First described by Freudenberger in 1974, it refers to fatigue or frustration stemming from prolonged stress and unmet expectations [26]. Burnout involves an imbalance between personal needs, values, and work demands [27,28] and comprises three main components: emotional exhaustion (a loss of energy or fatigue, manifesting physically or psychologically), depersonalization (negative, indifferent behaviors toward patients), and a reduced sense of personal achievement (feeling unfulfilled at work) [29,30].

Despite its significance, the literature lacks comparative analyses of occupational burnout in psychiatric versus somatic hospitals. Most studies focus on one type of hospital. It is widely believed that psychiatric nurses face higher stress and burnout due to their job’s nature. To address this knowledge gap, a study was conducted to compare stress and burnout levels in nurses from two hospitals within the same city—one somatic and one psychiatric.

The aim of our study was to report on the prevalence of stress and burnout among surveyed nurses employed at a psychiatric hospital and a somatic hospital. Based on the main objective, the following detailed research objectives were formulated:

1. How do respondents perceive the level of stress in their workplace?
2. Is occupational burnout prevalent among the surveyed nurses?
3. Do socio-demographic factors influence the levels of stress and occupational burnout?
4. Does the type of hospital where respondents are employed impact their levels of stress and job burnout?

## 2. Materials and Methods

### 2.1. Research Design

In the present study, two state hospitals in Poland participated in the survey: a somatic hospital and a psychiatric hospital. The survey was conducted between February and April 2024 among nurses who were actively working. Each nurse provided informed consent to participate in the study, and the survey questionnaire was designed to ensure anonymity, containing no sensitive data that could identify the respondents.

### 2.2. Research Tools

The research tool was a survey questionnaire containing questions on socio-demographic data and two standardized tools: the Mini-COPE questionnaire (Inventory for Measuring Coping with Stress) and MBI (The Maslach Burnout Inventory).

#### 2.2.1. Mini-COPE—Inventory for Measuring Coping with Stress

The Mini-COPE Inventory, developed by CH.S. Carver, is an abbreviated version of the Multidimensional Inventory for Measuring Coping with Stress, also known as COPE (The Coping Orientations to Problems Experienced). The Mini-COPE is designed for surveying both healthy and ill adults. The Polish adaptation of this tool [31] consists of 28 statements that represent 14 coping strategies, including active coping, planning, positive reevaluation, acceptance, humor, turning to religion, seeking emotional support, seeking instrumental support, distraction, denial, venting, using psychoactive substances, withdrawing from activities, and self-blame. For each statement, the respondent selects one of four possible responses, which are scored as follows: “I almost never do this” (0 points), “I rarely do this” (1 point), “I often do this” (2 points), and “I almost always do this” (3 points). Each of the 14 coping strategies is scored individually, with higher scores indicating more frequent use of a particular strategy. The tool is primarily used to measure dispositional coping, assessing typical reactions and feelings in situations of significant stress. The Polish version of Mini-COPE has demonstrated satisfactory psychometric properties. The internal consistency of the tool was assessed using the half-method, yielding a score of 0.86 (Guttman index: 0.87).

#### 2.2.2. MBI—The Maslach Burnout Inventory

The Maslach Burnout Inventory (MBI), developed by Maslach and Jackson [27] and adapted into Polish by Pasikowski [32], is used to assess the level of occupational burnout. This questionnaire consists of 22 statements that describe the psychophysical state of the respondent related to the biological determinants of stress experienced at work [33] and are divided into three categories corresponding to the key aspects of occupational burnout: emotional exhaustion, depersonalization, and reduced sense of personal achievement. Responses are given on a 7-point frequency scale, where 0 means “never” and 6 means “every day”. The score is calculated separately for each subscale by summing the points for each aspect: emotional exhaustion is categorized as high (>27), moderate (17–26), or low (0–16); depersonalization is categorized as high (>13), moderate (7–12), or low (0–6); and lack of achievement is categorized as high (0–31), moderate (32–38), or low (>39). Higher scores on the emotional exhaustion and depersonalization scales indicate more intense burnout, while lower scores on the sense of personal achievement scale are associated with higher burnout levels.

### 2.3. Participants

The study group consisted of a total of 379 nurses, with 189 employed at a somatic hospital and 190 employed at a psychiatric hospital. The inclusion criteria were full-time employment as a nurse in a psychiatric unit for more than one year and consent to participate in the study. It was planned to survey 200 nurses at each hospital who were working at the time of the survey, representing approximately 50% of all employed nurses at each hospital. After obtaining permission to conduct the survey, paper questionnaires were placed in the secretariats of the departments with a request for distribution to the nurses, to be returned in a sealed envelope once completed. A total of 400 survey questionnaires were distributed, and 384 were returned. After final verification, 379 correctly completed questionnaires were subjected to statistical analysis. In the somatic hospital, 94.5% of the distributed questionnaires were completed, and in the psychiatric hospital, 95.0% were completed.

### 2.4. Statistical Analysis

A chi-square test of independence was used to assess the significance of differences. To compare the significance of differences in the use of coping strategies (Mini-COPE measurements) between the two groups, the Mann–Whitney U test was used for detailed measurements, and the *t*-test was applied for groups of strategies (due to their proximity to normal distributions). The Cramer’s V coefficient was used to assess the level of relationship between two nominal variables, at least one of which had more than two values. During these analyses, the corresponding *p*-values were also calculated using the Monte Carlo method. The IBM SPSS 26.0 package and the Exact Tests module were used to conduct the analysis. Correlations were performed using Spearman’s rho coefficient. To facilitate interpretation, statistically significant results were marked with the convention *p* < 0.05.

### 2.5. Ethical Procedure

The participation of nurses in the study was voluntary and anonymous. The study was conducted in accordance with the ethical standards set forth in the Declaration of Helsinki (64th WmA General Assembly, Fortaleza, Brazil, October 2013) and in accordance with Polish legal regulations. The application was favorably approved by the Bioethics Committee of the State Academy of Applied Sciences in Przemyśl (KB/1/2024).

## 3. Results

### 3.1. Results of the Author’s Survey Questionnaire

Socio-demographic and occupational data collected in the survey included gender, age, length of service, type of hospital where respondents were employed, and whether they worked shifts. A total of 379 nurses participated in the survey, with 189 employed at a somatic hospital and 190 employed at a psychiatric hospital. All socio-demographic and professional data were self-reported. Among the respondents in the somatic hospital, 92.9% were women, compared to 83.2% in the psychiatric hospital. The remaining respondents were men, but due to the small size of this group, these socio-demographic data were not considered in the statistical analysis. The participants were nurses of various ages. A larger proportion of the youngest respondents and those over 55 were found in the group working in a hospital for somatically ill patients compared to the group working in a psychiatric hospital. The relationship between these variables was statistically significant (*p* < 0.05), but the strength of the relationship was found to be insignificant (Cramer’s V = 0.184) (Table 1).

Nurses who had been working full-time in psychiatric wards for more than a year, regardless of their position, participated in the study. The shortest and the longest length of service are more inclusive of the respondents in the group employed at a hospital for somatically ill patients. The correlation coefficient is statistically significant (*p* < 0.05), but the strength of the relationship was found to be insignificant (Cramer’s V = 0.158) (Table 2).

A total of 14.2% of the nurses surveyed had graduated from high school or medical college, 52.5% had a bachelor’s degree in nursing (i.e., completed their first degree), and 33.2% of the respondents held a master’s degree in nursing. There was no statistically significant relationship (*p* > 0.05) between place of work and degree of education (Table 3).

The nurses surveyed worked in different shift systems: 19.8% worked a single shift, 3.4% worked an 8 h shift, and 76.8% worked a 12 h shift. These shift systems included night shifts, as well as work on Sundays and holidays. A shift system with 12 h duties is more common for those working in a hospital for the mentally ill than for those working in a hospital for the somatically ill. The relationship between the variables is statistically significant (*p* < 0.05) and has a weak strength of association (Cramer’s V = 0.126) (Table 4).

### 3.2. Results of the Mini-COPE and MBI Questionnaires

Among the respondents employed at the somatic hospital, the average MBI burnout score was 48.54 ± 10.00, with the minimum level of burnout being 22 points and the maximum reaching 83 points. The subscale scores were as follows: emotional exhaustion 21.74 ± 5.43, depersonalization 10.63 ± 2.86, and a reduced sense of personal achievement 16.37 ± 4.06.

For respondents employed at the psychiatric hospital, the average burnout score was 48.56 ± 10.63. The minimum level of burnout in this group was the same as that of the somatic hospital respondents. The subscale scores were: emotional exhaustion 21.95 ± 6.08, depersonalization 10.24 ± 3.00, and a reduced sense of personal achievement 16.38 ± 4.78.

Analysis using the Mann–Whitney U test showed that the workplace did not statistically significantly differentiate the results of occupational burnout or the ways of coping with stress (*p* > 0.05), according to the Mini-COPE questionnaire. Detailed results are presented in Table 5.

Since statistical analysis did not show that workplace did not statistically significantly differentiate the results of job burnout and ways of coping with stress, another correlation was performed collectively for all respondents.

Analysis with the Kruskal–Wallis test showed that only the scores of depersonalization, active coping and support seeking varied statistically significantly by work system (*p* < 0.05). Respondents working in shifts are characterized by slightly higher depersonalization scores compared to respondents working in single shifts. In addition, respondents working in single-shift and shift work (12 h on-call) are more actively coping with stress and seeking support than respondents working in shift work (8 h on-call) (Table 6).

### 3.3. Correlation Results

In the group of respondents working at the somatic hospital, more than a dozen statistically significant correlations (*p* < 0.05) were found between occupational burnout and stress-coping strategies. However, only one correlation showed a clear strength of association. It was observed that as the scores for reduced sense of personal achievement increased, the level of helplessness also increased (Spearman’s rho = 0.332). Additionally, there were clear strengths of the relationship indicating that with greater overall professional burnout, the level of active coping and support seeking decreased (Table 7).

Among those working in a psychiatric hospital, compared to those in a somatic hospital, there were more statistically significant correlations between occupational burnout and ways of coping with stress (*p* < 0.05). The most pronounced relationships showed that higher levels of occupational exhaustion were associated with lower levels of active coping (Spearman’s rho = −0.317) and support seeking (Spearman’s rho = −0.352). Additionally, with a reduced sense of personal accomplishment, active coping scores decreased (Spearman’s rho = −0.326). Less pronounced relationships indicated that greater emotional exhaustion and a more reduced sense of personal accomplishment were associated with lower levels of support seeking (Table 8).

Eight statistically significant correlations (*p* < 0.05) were found between age, seniority, education, and the level of burnout and ways of coping with stress in the group working in a hospital for somatically ill patients, with weak strengths of association. It was observed that as age and seniority increase, depersonalization, turning to religion, and support seeking decrease. Additionally, those with longer work experience were more emotionally exhausted and engaged more actively in coping with stress (Table 9). 

In the group of people working in a hospital for the mentally ill, five statistically significant correlations (*p* < 0.05) were found between age, seniority, education, and the level of burnout and ways of coping with stress, with one showing a clear strength of association. Emotional exhaustion increased with longer job tenure (Spearman’s rho = 0.336). Weaker relationships indicated that older respondents had higher levels of emotional exhaustion and overall MBI burnout. Additionally, with longer tenure, levels of burnout increased and support seeking decreased (Table 10).

## 4. Discussion

The purpose of our study was to examine the prevalence of stress and occupational burnout among nurses employed in a psychiatric hospital and a somatic hospital. The results showed that the place of employment does not statistically significantly differentiate levels of occupational burnout or stress-coping strategies, as the results were almost identical.

Analyzing the literature, the authors did not find studies addressing these issues jointly. Existing studies typically focus on either psychiatric nurses or those employed in hospitals for somatic patients. The commonly held belief that psychiatric nurses are more prone to stress and occupational burnout was not confirmed in our study. 

Ghavidel et al. claim that nursing patients diagnosed with mental illness is stressful due to interpersonal relationships within the treatment team and constant contact with individuals with complex emotional demands [21]. These stressful working conditions may place nurses working in psychiatric hospitals at greater risk of professional burnout [34]. Newman et al. found that most nurses experienced moderate occupational burnout, although they remained confident in their practice and found their work rewarding. However, they reported feeling stressed due to workload and conflicts within the treatment team [35]. Occupational burnout, stress, sleep quality, and depressive symptoms may form a complex relationship of mutual influence in the nursing profession [36], as confirmed by other studies [23,25,37].

Tununu et al. observed low levels of occupational burnout among respondents, with high job satisfaction. Support staff reported significantly higher levels of emotional exhaustion than psychiatric nurses and graduate nurses. Seniority impacted depersonalization scores, although burnout in all three domains was not present [38]. Another self-reported study indicated that emotional exhaustion increases among those working longer in mental hospitals. Older respondents exhibited higher levels of emotional exhaustion and overall MBI burnout. Additionally, with longer tenure, the level of burnout increased while support-seeking behaviors decreased. For nurses in hospitals for somatic patients, as age and seniority increased, depersonalization levels rose, while reliance on religion and support seeking declined. Emotional exhaustion also increased, along with active stress-coping efforts.

Alenezi et al. conducted a survey among nurses at a Saudi hospital for the mentally ill, reporting high levels of emotional exhaustion, depersonalization, and low personal achievement, indicating a high overall level of job burnout [39]. Konstantinou et al. studied Greek psychiatric nurses and found high emotional exhaustion, moderate depersonalization, and low personal achievement, resulting in medium to high burnout levels [40]. Other studies on occupational burnout among nurses working with mentally ill patients have reported moderate to low levels across all domains, differing from the results of the current study [41], a finding supported by other research [42,43].

A study of nurses in intensive care units revealed that the work environment is a significant factor in stress and burnout, with moderate burnout levels observed [44]. Similar results were found among oncology nurses [45]. Burnout in nurses is associated with reduced safety and quality of care, lower patient satisfaction, and decreased organizational commitment and productivity. While traditionally seen as an individual issue, addressing burnout at the organizational level offers a broader perspective on this phenomenon [46].

### Limitations of the Study

A strength of the study was that it surveyed most of the nursing staff employed at the hospitals. However, a notable limitation is that it was conducted at only one somatic hospital and one psychiatric hospital within the same city. Furthermore, the possibility of nurses exchanging opinions during the survey may have influenced responses. Future research should include more healthcare units providing similar services to validate or refute the findings.

## 5. Conclusions

Respondents working in both psychiatric and somatic hospitals rate the occurrence of occupational stress at a moderate level. The place of work does not statistically significantly differentiate the results of occupational burnout or ways of coping with stress. Among nurses employed in a somatic hospital, as age and length of service increase, depersonalization, turning to religion, and seeking support in situations of increased stress increase. In addition, those who have been working longer are more emotionally exhausted and more actively coping with stress. In contrast, levels of emotional exhaustion and overall MBI burnout increase among psychiatric nurses with longer tenure. 

The results presented here indicate that managers should mobilize nursing staff to participate in on-the-job training programs that address stress management in order to acquire the necessary skills to cope with stress at work.

## Figures and Tables

**Table 1:** Age distribution of surveyed nurses by place of employment.

Variable			Place of Employment		Total
			Somatic Hospital	Psychiatric Hospital	
Age (years)	Below 25	%	16.4%	9.5%	12.9%
	26–35	%	24.9%	24.2%	24.5%
	36–45	%	13.2%	25.3%	19.3%
	46–55	%	26.5%	27.9%	27.2%
	Above 55	%	19.0%	13.2%	16.1%
Total		%	100.0%	100.0%	100.0%
Cramer’s V	0.184	12.775	4	0.012	0.011
coefficient	value	Chi-square	df	*p*	Monte Carlo *p*

**Table 2:** Length of service of surveyed nurses by place of employment.

Variable			Place of Employment		Total
			Somatic Hospital	Psychiatric Hospital	
Length of service (years)	Below 5	%	29.1%	26.8%	28.0%
	5–10	%	14.8%	15.8%	15.3%
	11–20	%	13.8%	19.5%	16.6%
	21–30	%	18.0%	24.2%	21.1%
	Above 30	%	24.3%	13.7%	19.0%
Total		N	189	190	379
		%	100.0%	100.0%	100.0%
Cramer’s V	0.158	9.494	4	0.050	0.049
coefficient	value	Chi-square	df	*p*	Monte Carlo *p*

**Table 3:** Education of surveyed nurses by place of employment.

Variable			Place of Employment		Total
			Somatic Hospital	Psychiatric Hospital	
Education	High school Vocational school	%	18.0%	10.5%	14.2%
	Bachelor’s	%	51.3%	53.7%	52.5%
	Master’s	%	30.7%	35.8%	33.2%
Total		N	189	190	379
		%	189	190	379
Cramer’s V	0.110	Chi-square	df	*p*	Monte Carlo *p*
coefficient	value				

**Table 4:** Work system of surveyed nurses by place of employment.

Variable			Place of Employment		Total
			Somatic Hospital	Psychiatric Hospital	
Work system	Single shift (7 h 35 m)	%	24.3%	15.3%	19.8%
	Multiple shifts (8 h)	%	4.2%	2.6%	3.4%
	Multiple shifts (12 h)	%	71.4%	82.1%	76.8%
Total		N	189	190	379
		%	100.0%	100.0%	100.0%
Cramer’s V	0.126	6.059	2	0.048	0.050
coefficient	value	Chi-square	df	*p*	Monte Carlo *p*

**Table 5:** Mini-COPE and MBI questionnaire scores by type of hospital where respondents were employed.

Workplace		MBI (22–88 pts.)	Emotional Exhaustion (9–36 pts.)	Depersonalization (5–20 pts.)	Reduced Sense of Personal Achievement (8–32 pts.)	Active Coping	Helplessness	Seeking Support	Avoidance Behavior	Turn to Religion	Acceptance	Sense of Humor
Somatic hospital	Mean	48.74	21.74	10.63	16.37	1.88	0.90	1.78	1.36	1.53	1.82	1.01
	Median	49.00	22.00	11.00	16.00	2.00	0.83	1.75	1.33	1.50	2.00	1.00
	Average rank	192.52	187.91	199.67	194.14	190.01	200.35	196.44	198.32	198.13	192.64	185.69
	n	189	189	189	189	189	189	189	189	189	189	189
	Standard deviation	9.30	5.43	2.86	4.06	0.62	0.49	0.65	0.52	0.87	0.76	0.76
	Minimum	22.00	9.00	5.00	8.00	0.33	0.00	0.00	0.17	0.00	0.00	0.00
	Maximum	73.00	36.00	20.00	27.00	3.00	2.33	3.00	2.67	3.00	3.00	3.00
Psychiatric hospital	Mean	48.56	21.95	10.24	16.38	1.91	0.81	1.71	1.29	1.40	1.78	1.04
	Median	48.00	22.00	10.00	16.00	2.00	0.83	1.75	1.33	1.50	2.00	1.00
	Average rank	187.49	192.08	180.38	185.88	189.99	179.70	183.59	181.72	181.91	187.38	194.29
	n	190	190	190	190	190	190	190	190	190	190	190
	Standard deviation	10.63	6.08	3.00	4.78	0.57	0.51	0.70	0.50	0.98	0.74	0.71
	Minimum	22.00	9.00	5.00	8.00	0.00	0.00	0.00	0.00	0.00	0.00	0.00
	Maximum	83.00	36.00	19.00	32.00	3.00	2.17	3.00	3.00	3.00	3.00	3.00
Total	Mean	48.65	21.84	10.44	16.37	1.89	0.86	1.74	1.32	1.47	1.80	1.03
	Median	49.00	22.00	10.00	16.00	2.00	0.83	1.75	1.33	1.50	2.00	1.00
	Average rank	379	379	379	379	379	379	379	379	379	379	379
	n	9.98	5.76	2.93	4.43	0.60	0.50	0.68	0.51	0.92	0.74	0.73
	Standard deviation	22.00	9.00	5.00	8.00	0.00	0.00	0.00	0.00	0.00	0.00	0.00
	Minimum	83.00	36.00	20.00	32.00	3.00	2.33	3.00	3.00	3.00	3.00	3.00
Mann–Whitney U		Max.	17,560	16,127	17,173	17,954	15,998	16,738	16,382	16,417	17,456	17,140
*p*		0.654	0.711	0.085	0.462	0.999	0.065	0.250	0.138	0.144	0.631	0.434
*p* (Monte Carlo)		0.649	0.713	0.087	0.466	1.000	0.063	0.251	0.138	0.138	0.624	0.438

**Table 6:** Mini-COPE and MBI questionnaire scores by work system of respondents.

Work System		MBI (22–88 pts.)	Emotional Exhaustion (9–36 pts.)	Depersonalization (5–20 pts.)	Reduced Sense of Personal Achievement (8–32 pts.)	Active Coping	Helplessness	Seeking Support	Avoidance Behavior	Turn to Religion	Acceptance	Sense of Humor
Single shift—7 h 35 m	Mean	46.96	21.13	9.65	16.18	1.99	0.85	1.89	1.32	1.52	1.77	1.12
	Median	47.00	20.00	10.00	16.00	2.00	0.83	2.00	1.33	1.50	2.00	1.00
	Average rank	173.84	176.35	161.12	191.35	214.12	191.67	215.26	194.96	200.75	187.66	203.58
	n	78	78	78	78	78	78	78	78	78	78	78
	Standard deviation	9.83	5.97	2.79	4.21	0.59	0.45	0.61	0.47	0.93	0.70	0.78
	Minimum	22.00	9.00	5.00	8.00	0.33	0.00	0.50	0.17	0.00	0.00	0.00
	Maximum	71.00	36.00	20.00	26.00	3.00	2.33	3.00	2.33	3.00	3.00	3.00
Multiple shifts—8 h	Mean	47.36	21.07	10.50	15.79	1.54	0.76	1.39	1.19	1.21	1.75	0.96
	Median	49.00	21.50	10.00	14.00	1.67	0.83	1.63	1.42	1.25	2.00	1.00
	Average rank	184.18	187.11	182.57	161.14	139.00	178.54	142.21	169.75	162.64	189.39	189.96
	n	14	14	14	14	14	14	14	14	14	14	14
	Standard deviation	9.01	6.50	4.05	5.95	0.82	0.46	0.71	0.58	1.03	0.67	0.60
	Minimum	27.00	9.00	5.00	10.00	0.00	0.00	0.00	0.33	0.00	0.50	0.00
	Maximum	62.00	33.00	18.00	32.00	2.83	1.50	2.50	2.33	3.00	2.50	2.00
Multiple shifts—12 h	Mean	49.02	21.96	10.61	16.45	1.88	0.86	1.72	1.32	1.45	1.81	1.01
	Median	49.00	22.00	10.00	16.00	2.00	0.83	1.75	1.33	1.50	2.00	1.00
	Average rank	197.88	197.07	201.36	194.31	189.29	193.39	188.83	192.93	191.73	193.94	189.66
	n	292	292	292	292	292	292	292	292	292	292	292
	Standard deviation	10.08	5.71	2.89	4.43	0.59	0.51	0.69	0.52	0.92	0.76	0.72
	Minimum	22.00	9.00	5.00	8.00	0.00	0.00	0.00	0.00	0.00	0.00	0.00
	Maximum	83.00	36.00	20.00	32.00	3.00	2.33	3.00	3.00	3.00	3.00	3.00
Total	Mean	48.54	21.76	10.41	16.37	1.89	0.85	1.74	1.32	1.46	1.80	1.03
	Median	49.00	22.00	10.00	16.00	2.00	0.83	1.75	1.33	1.50	2.00	1.00
	Average rank	379	379	379	379	379	379	379	379	379	379	379
	n	10.00	5.79	2.94	4.44	0.60	0.50	0.68	0.51	0.93	0.74	0.73
	Standard deviation	22.00	9.00	5.00	8.00	0.00	0.00	0.00	0.00	0.00	0.00	0.00
	Minimum	83.00	36.00	20.00	32.00	3.00	2.33	3.00	3.00	3.00	3.00	3.00
Kruskal–Wallis H		Maximum	2.19	8.31	1.21	6.52	0.25	6.57	0.64	1.50	0.22	1.02
*p*		0.226	0.335	0.016	0.546	0.038	0.884	0.038	0.727	0.473	0.896	0.600
*p* (Monte Carlo)		0.226	0.330	0.016	0.548	0.035	0.890	0.036	0.726	0.474	0.899	0.591

**Table 7:** Correlations between Mini-COPE and MBI—nursing staff employed in a somatic hospital (n = 189).

Workplace = Somatic Hospital			MBI (22–88 pts.)	Emotional Exhaustion (9–36 pts.)	Depersonalization (5–20 pts.)	Reduced Sense of Personal Achievement (8–32 pts.)
Spearman’s rho	Active coping	Correlation coefficient	−0.297	−0.199	−0.138	−0.260
	Helplessness	Correlation coefficient	0.224	0.068	0.102	0.332
	Seeking support	Correlation coefficient	−0.287	−0.266	−0.186	−0.164
	Avoidance behavior	Correlation coefficient	0.011	−0.067	−0.009	0.171
	Turn to religion	Correlation coefficient	−0.099	−0.038	−0.143	−0.070
	Acceptance	Correlation coefficient	−0.107	−0.066	−0.026	−0.105
	Sense of humor	Correlation coefficient	0.045	−0.066	−0.034	0.188

**Table 8:** Correlations between Mini-COPE and MBI—nursing staff employed in a psychiatric hospital (n = 190).

Workplace = Psychiatric Hospital			MBI (22–88 pts.)	Emotional Exhaustion (9–36 pts.)	Depersonalization (5–20 pts.)	Reduced Sense of Personal Achievement (8–32 pts.)
Spearman’s rho	Active coping	Correlation coefficient	−0.317	−0.183	−0.180	−0.326
	Helplessness	Correlation coefficient	0.275	0.211	0.174	0.205
	Seeking support	Correlation coefficient	−0.352	−0.286	−0.204	−0.283
	Avoidance behavior	Correlation coefficient	−0.050	−0.074	−0.091	0.011
	Turn to religion	Correlation coefficient	−0.073	−0.004	−0.144	−0.082
	Acceptance	Correlation coefficient	−0.196	−0.099	−0.154	−0.181
	Sense of humor	Correlation coefficient	−0.095	−0.154	0.015	−0.081

**Table 9:** Correlations between socio-demographic data and Mini-COPE and MBI—nursing staff employed in a hospital for somatic patients (n = 189).

Workplace = Somatic Hospital		Age	Job Seniority	Education
MBI (22–88 pts.)	Correlation coefficient	0.054	0.094	−0.023
Emotional exhaustion (9–36 pts.)	Correlation coefficient	0.132	0.159	−0.057
Depersonalization (5–20 pts.)	Correlation coefficient	0.158	0.192	−0.104
Reduced sense of personal achievement (8–32 pts.)	Correlation coefficient	−0.142	−0.119	0.124
Active coping	Correlation coefficient	0.115	0.144	−0.007
Helplessness	Correlation coefficient	−0.065	−0.061	−0.045
Seeking support	Correlation coefficient	−0.239	−0.197	−0.043
Avoidance behavior	Correlation coefficient	−0.137	−0.105	0.005
Turn to religion	Correlation coefficient	0.213	0.242	−0.069
Acceptance	Correlation coefficient	0.083	0.094	0.051
Sense of humor	Correlation coefficient	−0.120	−0.109	0.042

**Table 10:** Correlations between socio-demographic data and Mini-COPE and MBI—nursing staff employed in a psychiatric hospital (n = 190).

Workplace = Psychiatric Hospital		Age	Job Seniority	Education
MBI (22–88 pts.)	Correlation coefficient	0.146	0.172	0.022
Emotional exhaustion (9–36 pts.)	Correlation coefficient	0.292	0.336	0.054
Depersonalization (5–20 pts.)	Correlation coefficient	0.006	0.025	0.102
Reduced sense of personal achievement (8–32 pts.)	Correlation coefficient	−0.053	−0.038	−0.020
Active coping	Correlation coefficient	0.070	0.042	−0.032
Helplessness	Correlation coefficient	0.007	−0.037	0.126
Seeking support	Correlation coefficient	−0.099	−0.146	0.018
Avoidance behavior	Correlation coefficient	0.013	−0.058	0.057
Turn to religion	Correlation coefficient	0.088	0.058	−0.013
Acceptance	Correlation coefficient	−0.009	−0.022	−0.090
Sense of humor	Correlation coefficient	−0.085	−0.107	−0.069

## Data Availability

The datasets used and analyzed during the current study available from the corresponding author on reasonable request.
